# Risk of febrile neutropenia among patients with multiple myeloma or lymphoma who undergo inpatient versus outpatient autologous stem cell transplantation: a systematic review and meta-analysis

**DOI:** 10.1186/s12885-018-5054-6

**Published:** 2018-11-16

**Authors:** Weerapat Owattanapanich, Kittima Suphadirekkul, Chutima Kunacheewa, Patompong Ungprasert, Kannadit Prayongratana

**Affiliations:** 1 Division of Hematology, Department of Medicine, Faculty of Medicine Siriraj Hospital, Mahidol University, 2 Wanglang Road, Bangkoknoi, Bangkok, 10700 Thailand; 2 Division of Medicine, Thonburi Hospital, Bangkok, 10700 Thailand; 3 Clinical Epidemiology Unit, Department of Research and Development, Faculty of Medicine Siriraj Hospital, Mahidol University, Bangkok, 10700 Thailand; 4 Division of Hematology, Department of Medicine, Phramongkutklao Hospital and College of Medicine, Bangkok, 10400 Thailand

**Keywords:** Outpatient, Autologous, Stem cell transplantation, Multiple myeloma, Lymphoma

## Abstract

**Background:**

Outpatient autologous stem cell transplantations (ASCTs) in multiple myeloma and lymphoma patients have been shown to reduce the overall costs and improve the quality of life relative to inpatient ASCTs. This systematic review and meta-analysis was performed with the aim of comprehensively comparing the risk of febrile neutropenia developing in ASCT outpatients and inpatients who have multiple myeloma or lymphoma.

**Methods:**

To be eligible for the meta-analysis, studies needed to be either randomized, controlled studies or cohort studies. They also need to have two groups of patients with multiple myeloma or lymphoma who underwent ASCT, with the treatment being provided to one group in an outpatient setting and to the other on an inpatient basis. The studies had to report our primary outcome of interest, the rate of febrile neutropenia after stem cell infusion, for both groups. The Mantel–Haenszel method was used to pool the effect estimates and 95% confidence intervals of each study.

**Results:**

From 9 eligible studies, a total of 1940 patients were included in the meta-analysis. Contrary to conventional concerns, the patients who underwent the outpatient ASCT had a significantly lower risk of developing febrile neutropenia than those admitted for ASCT, with a pooled odds ratio (OR) of 0.44 (95% confidence interval [CI]: 0.29–0.65; *p* < 0.0001; I^2^ = 52%). The risk of septicemia was also significantly lower for the outpatients than the inpatients, with a pooled OR of 0.40 (95% CI: 0.16–0.97; *p* = 0.04; I^2^ = 23%). Additional analyses found that the odds of having grade 2–3 mucositis and transplant-related mortality were numerically lower for the outpatient group, although the pooled result was not statistically significant. The odds of surviving at 2–3 years was also numerically higher for the ASCT outpatients, but the difference did not reach statistical significance.

**Conclusions:**

This study found a significantly lower odds of developing febrile neutropenia and septicemia among patients with multiple myeloma and lymphoma who received an outpatient ASCT than among those who had an inpatient ASCT.

**Electronic supplementary material:**

The online version of this article (10.1186/s12885-018-5054-6) contains supplementary material, which is available to authorized users.

## Background

Autologous stem cell transplantation (ASCT) is a major therapeutic option for patients with multiple myeloma who are eligible for transplantation and achieved at least a partial response after combination chemotherapy [1–4]. The main objectives of ASCT are to eliminate residual clonal plasma cells and to produce a deeper response, which would improve the overall prognosis by providing a better progression-free survival and overall survival (OS) rate [4–7]. On the other hand, the use of ASCT in patients with lymphoma is limited to those with relapse or refractory disease, and to those with lymphoma subtypes that have a poor long-term response after chemotherapy (such as peripheral T-cell lymphoma, mantle cell lymphoma, and primary CNS lymphoma) [8–11].

The major disadvantages of ASCT include its higher rate of short-term complications, the requirement for hospitalization, and the associated costs [12, 13]. In 1993, an outpatient ASCT program was developed with the aims of reducing the hospitalization expenses and improving patients’ quality of life [14, 15]. A cost-benefit analysis undertaken by a Canadian study estimated that the total cost of an ASCT in multiple myeloma patients was 42,723 Canadian dollars per outpatient, compared with 62,259 Canadian dollars per inpatient [16]. Another study found that patients treated with ASCT without hospitalization reported a greater social and family well-being than those treated in hospital [17]. Nevertheless, outpatient ASCTs are still not widely utilized due to concerns about the risk of infection in the absence of the protective isolation practices employed during hospitalization. The current systematic review and meta-analysis was performed to comprehensively compare the risks of developing febrile neutropenia in the two strategies.

## Methods

### Data sources and searches

Published studies indexed in the MEDLINE and EMBASE databases as at August 1, 2018 were independently searched by two investigators (W.O. and K.S.) using a search strategy that included the terms “autologous stem cell transplantation” and “outpatient”. Additional file 1: Data 1 illustrates the search strategy. An effort to identify additional eligible studies was made by reviewing the references of the included studies. This study was undertaken in accordance with the Preferred Reporting Items for Systematic Reviews and Meta-Analyses statement, which is available as Additional file 2: Data 2 [18].

### Selection criteria and data extraction

To be eligible for the meta-analysis, studies needed to be randomized, controlled studies or cohort studies (either prospective or retrospective). They also needed to have two groups of patients with either multiple myeloma or lymphoma who underwent ASCT, with one group receiving the treatment in an outpatient setting and the other as inpatients. For both groups, the studies had to report our primary outcome of interest: the rate of febrile neutropenia after the stem cell infusion. The secondary outcomes of interest were the rate of septicemia, *Clostridioides (C.) difficile* infection, grade 2–3 mucositis, transplant-related mortality (TRM), and overall survival (OS). Although data on those items were also collected for analysis, they were not part of the inclusion criteria. Two investigators independently assessed the eligibility of each study, with disagreements resolved by discussion and consensus.

### Definitions of outcomes

Febrile neutropenia was defined as a single oral temperature of ≥38.3 °C (101 °F) or a temperature ≥ 38 °C (100.4 °F) over 1 h, and with either an absolute neutrophil count of < 0.5 × 10^9^ neutrophils/l or an absolute neutrophil count of < 1 × 10^9^ neutrophils/l that was predicted to decline to 0.5 × 10^9^ neutrophils/l over the following 48 h. Septicemia was defined as the presence of virulent microorganisms, especially bacteria, in the bloodstream. The OS rate was defined as the proportion of patients who were still alive at the time of interest. Finally, the TRM rate was defined as the proportion of patients who succumbed by day + 100 after undergoing the ASCT.

### Quality assessment

The Jadad quality assessment scale was used to assess the quality of the included randomized, controlled studies [19]. The quality of the included cohort studies was assessed using the Newcastle–Ottawa Scale. This 3-item scoring system evaluates the selection of research participants, the level of comparability between the groups, and the ascertainment of the outcome of interest [20].

### Statistical analysis

Review Manager software, version 5.3, from the Cochrane Collaboration (London, United Kingdom) was used to perform all statistical analyses. The Mantel–Haenszel method was used to pool the effect estimates and 95% confidence intervals from each study [21]. The statistical heterogeneity among the included studies was evaluated using Cochran’s Q test and quantified using the I^2^ statistic. The I^2^ values were classified as follows: 0–25% indicated insignificant heterogeneity; 26–50%, low heterogeneity; > 50% to ≤75%, moderate heterogeneity; and > 75%, high heterogeneity [22]. Because of the high likelihood of between-study heterogeneity, we employed the random-effects model rather than the fixed-effects model. A funnel plot was used to evaluate the publication bias. *P*-values less than 0.05 were considered statistically significant.

## Results

**Fig. 1:**
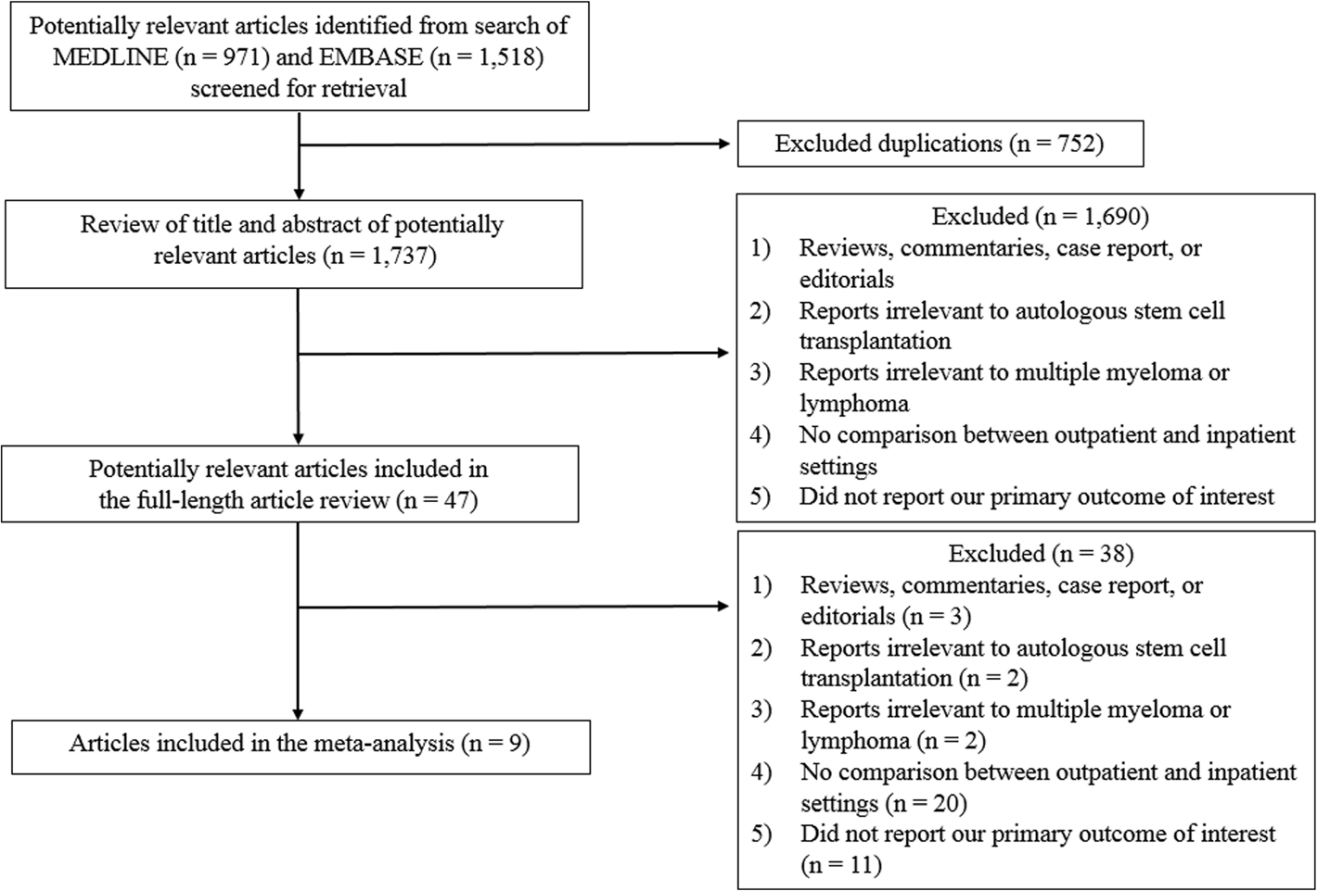
The literature review process

A total of 2489 potentially relevant articles were identified (971 from MEDLINE, and 1518 from EMBASE). Of those, 752 duplicated articles were excluded. The remaining 1737 articles were evaluated for relevance via a review of their titles and abstracts. At this stage, a total of 1690 articles were excluded for one or more of the following reasons: 1) type of article (reviews, commentaries, case reports, and editorials); 2) reports irrelevant to ASCT; 3) reports irrelevant to multiple myeloma or lymphoma; 4) no comparison of the outpatient and inpatient settings; and/or 5) did not report the outcome of interest. Of the remaining 47 articles that underwent a full-length article review, 38 were excluded for reasons similar to the first review round, leaving 9 studies (one prospective cohort study and eight retrospective cohort studies) for inclusion in the meta-analysis [23–31]. The literature review and selection process are illustrated in Fig. 1.

### Baseline patient characteristics

The nine eligible articles comprised 1940 patients (740 in the outpatient-ASCT arm and 1200 in the inpatient-ASCT arm). The outpatient age range was 17 to 78 years whereas that for the inpatients was 16 to 82 years. Males predominated in both groups. Using the Eastern Cooperative Oncology Group and Karnofsky Performance Status Scale, the performance status was good for the majority of patients in both groups. Over 80% of the patients had multiple myeloma, followed by lymphoma (12.5%) and other hematologic malignancies (0.9%).

### ASCT procedures and anti-microbial prophylaxis

Almost all cases received stem cells from peripheral blood sources, with comparable CD34+ doses being employed for the two groups. Several ASCT conditioning regimens were utilized. For patients with multiple myeloma, high-dose melphalan (100–200 mg/m^2^/day) for 2 days was most commonly used, whereas a combination of BCNU, etoposide, cytarabine, and melphalan (BEAM) was the most frequent regimen for lymphoma. Both the outpatient and inpatient group received anti-infective prophylaxes comprised of antiviral, antifungal, and antibacterial medications.

### Care programs for ASCT outpatients

The outpatient care programs tended to have the following characteristics: 1) the patients’ residences were close to the hospital (a 20- to 40-min drive from the center); 2) a caregiver was available 24 h a day; and 3) depending on the study, blood samples were taken every 1 to 3 days. However, a variety of management approaches were noted for the delivery of the conditioning regimens and stem cell infusions to the patients. In some of the included studies, all procedures were undertaken in an outpatient setting, whereas in the other studies, a short hospitalization was required for the administration of the conditioning regimen and stem cell infusion.

The baseline characteristics of the participants, study methodology, and quality assessment score for each study are summarized in Table 1. The details of the ASCT procedure, infectious prophylaxis, and outpatient management protocol employed by each study are available as Additional file 3: Table S1.

**Table 1:** Baseline patient characteristics of each included article

References	Group	No.	Sex (M/ F)	Median age (years, range)	HCT-CI	ECOG-PS/ KPS*	Diseases	Donor source	Median CD 34 + (× 10^6^ cells/kg, range)	Study period	Type	Quality assessment
Morabito 2002 [23]	OP	29	19/10	55.3	NR	NR	MM: 29	PBSC	5.3	1998–2001	R	Selection: 3 Comparability: 2 Outcome: 2
	IP	27	18/9	49.6	NR	NR	MM: 27	PBSC	6.4			
Ferna’ndez-Avilés 2006 [24]	OP	50	31/19	47 (20–65)	NR	0: 25, 1: 18, 2: 7	MM:13, Lym: 28, ALL: 4, AML: 1, CML: 2, CLL: 2	PBSC except 1 case was mixed source	3.2 (1.8–21)	2000–2005	R	Selection: 3 Comparability: 2 Outcome: 2
	IP	50	27/23	50 (20–68)	NR	0: 23, 1: 18, 2: 9	MM:13, Lym:28, ALL 3, AML: 2, CML: 2, CLL: 2	PBSC except 1 case was mixed source	2.7 (0.9–15)			
Martino 2015 [25]	OP	25	18/7	59 (42–65)	NR	NR	MM: 25	NR	5 (2.1–5.9)	NR	P	Selection: 4 Comparability: 2 Outcome: 2
	IP	33	20/13	62 (43–67)	NR	NR	MM 33	NR	4.9 (2.1–5.8)			
Graff 2015 [26]	OP	95	63/32	58 (20–76)	0–2: 57 ≥3: 38	≤80: 6, 90: 49, 100: 40	MM: 63, Lym: 32	NR	4.4 (1.9–12.9)	2009–2012	R	Selection: 3 Comparability: 2 Outcome: 3
	IP	135	86/49	59 (21–76)	0–2: 64 ≥3: 71	≤80: 36, 90: 72, 100: 27	MM: 88, Lym: 47	NR	4.6 (1.9–16.5)			
Paul 2015 [27]	OP	82	51/31	59 (28–71)	0–1: 82	NR	MM: 82	NR	NR	2003–2010	R	Selection: 3 Comparability: 2 Outcome: 2
	IP	219	128/91	60 (29–75)	0–1: 219	NR	MM: 219	NR	NR			
Reid 2016 [28]	OP	58	38/20	58 (17–72)	0–2: 46 ≥3: 12	NR	Lym: 58	PBSC	4.6 (2–16.8)	2011–2014	R	Selection: 4 Comparability: 2 Outcome: 2
	IP	49	30/19	59 (16–74)	0–2: 37 ≥ 3: 12	NR	Lym: 49	PBSC	4.2 (2–18.9)			
Abid 2017 [29]	OP	10	6/4	56 (39–62)	0–2: 10	0: 6, 1: 4, 2: 0	MM: 10	PBSC	9.2 (2.8–18.9)	2011–2015	R	Selection: 2 Comparability: 2 Outcome: 2
	IP	11	6/5	58 (45–64)	0–2: 9, ≥3: 2	0: 5, 1: 4, 2: 2	MM: 11	PBSC	8.5 (2.5–17.8)			
Lisenko 2017 [30]	OP	14	9/5	NR	NR	0–1: 14	MM: 14	PBSC	9.7 (7.4–24.8)	2012–2016	R	Selection: 3 Comparability: 2 Outcome: 2
	IP	**7**	6/1	NR	NR	0–1: 7	MM: 7	PBSC	13.7 (9.1–23)			
Shah 2017 [31]	OP	377	234/143	58 (34–78)	< 3: 207 ≥ 3: 170	90*	MM: 377	NR	NR	2008–2012	R	Selection: 3 Comparability: 2 Outcome: 2
	IP	669	369/300	62 (31–82)	< 3: 301 ≥ 3: 368	90*	MM: 669	NR	NR			

Abbreviations: *ALL* Acute lymphoblastic leukemia, *AML* Acute myeloid leukemia, *CLL* Chronic lymphocytic leukemia, *CML* Chronic myeloid leukemia, *ECOG-PS* Eastern Cooperative Oncology Group-Performance status, *F* Female, *HCT-CI* Hematopoietic Cell Transplantation-Comorbidity Index, *IP* Inpatient, *KPS* Kanofsky performance status, *Lym* Lymphoma, *M* Male, *MM* Multiple myeloma, *NR* Not reported, *OP* Outpatient, *P* Prospective cohort, *PBSC* Peripheral blood stem cell, *R* Retrospective cohort

### Risk of febrile neutropenia, septicemia, and *C. difficile* infection after ASCT

**Fig. 2:**
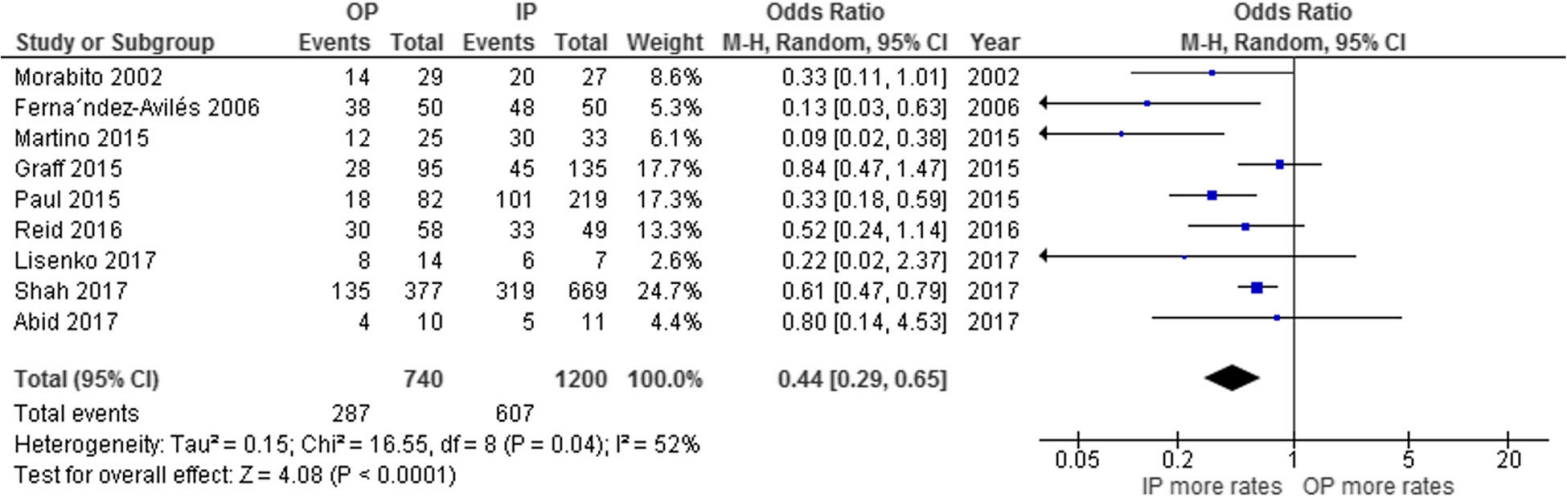
Forest plot of the analysis of the odds of developing febrile neutropenia in the outpatient versus inpatient autologous stem cell transplantation groups

**Fig. 3:**
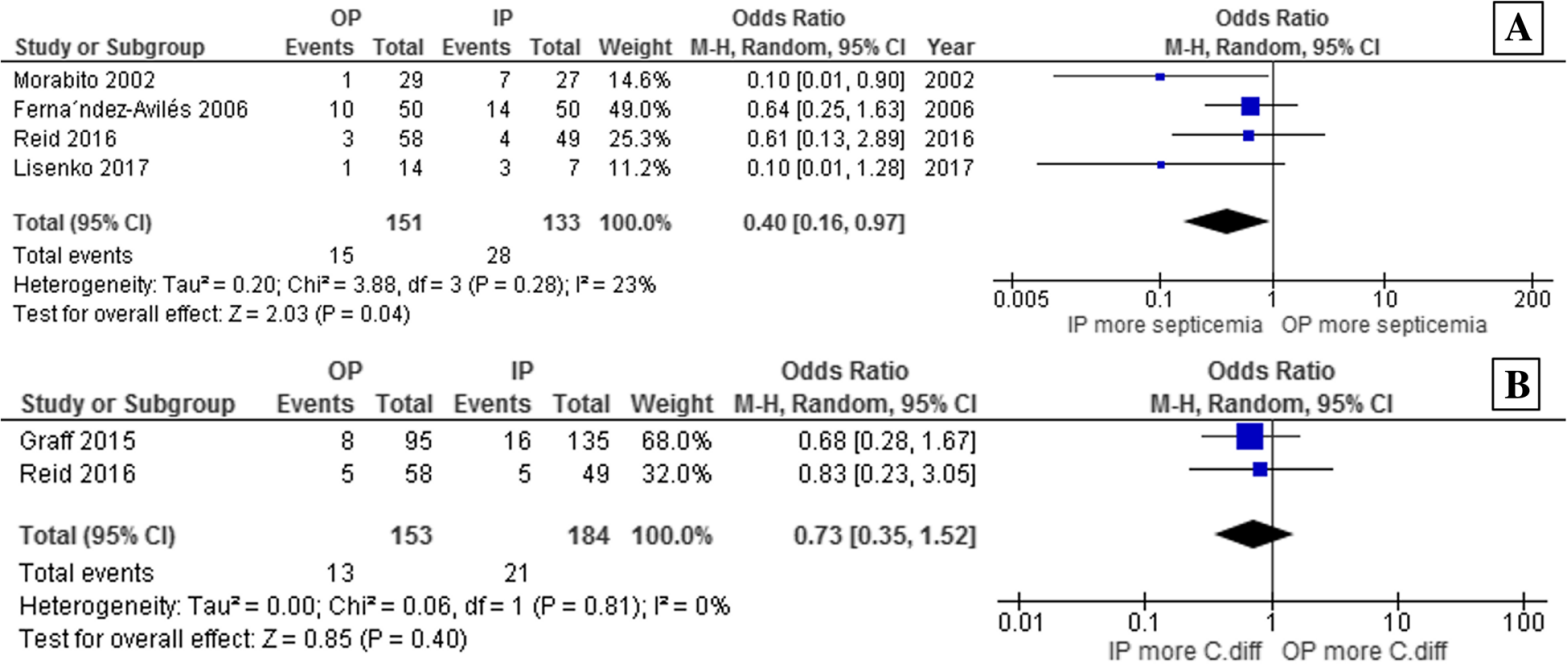
Forest plot of the analysis of the odds of developing septicemia (**a**) and *Clostridioides difficile* infection (**b**) in the outpatient versus inpatient autologous stem cell transplantation groups

Patients who underwent an outpatient ASCT had a significantly lower risk of developing febrile neutropenia than patients who were admitted for the ASCT, with a pooled odds ratio (OR) of 0.44 (95% confidence interval [CI]: 0.29–0.65; *p* < 0.0001; I^2^ = 52%; Fig. 2) [23–31]. The risk of septicemia was also significantly lower for the outpatients than the inpatients, with a pooled OR of 0.40 (95% CI: 0.16–0.97; *p* = 0.04; I^2^ = 23%; Fig. 3a) [23, 24, 28, 30]. However, the risk of *C. difficile* infection was not significantly different for the two patient groups, with a pooled OR of 0.73 (95% CI: 0.35–1.52; *p* = 0.40; I^2^ = 0%; Fig. 3b) [26, 28].

### Risk of non-infectious complications after ASCT

**Fig. 4:**
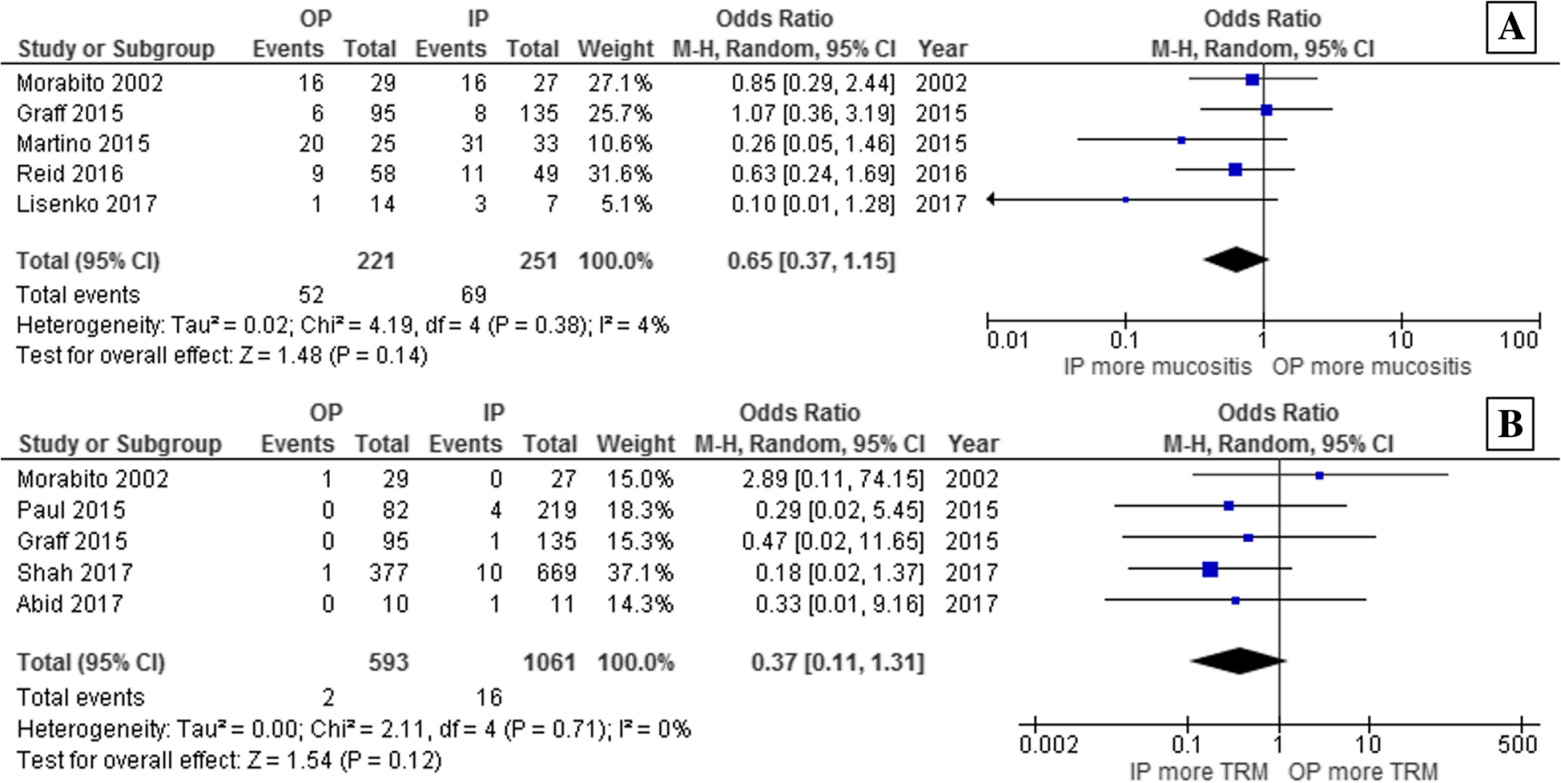
Forest plot of the analysis of the odds of developing (**a**) grade 2–3 mucositis and (**b**) transplant-related mortality in the outpatient versus inpatient autologous stem cell transplantation groups

While the odds of having grade 2–3 mucositis was numerically lower for the outpatient group, the pooled result was not statistically significant (pooled OR 0.65; 95% CI, 0.37–1.15; *p* = 0.14; I^2^ = 4%; Fig. 4a) [23, 25, 26, 28, 30]. Similarly, though the odds ratio of having a TRM appeared to be lower for the outpatient group, it did not achieve statistical significance (pooled OR 0.37; 95% CI, 0.11–1.31; *p* = 0.12; I^2^ = 0%; Fig. 4b) [23, 26, 27, 29, 31].

### Long-term outcomes

**Fig. 5:**
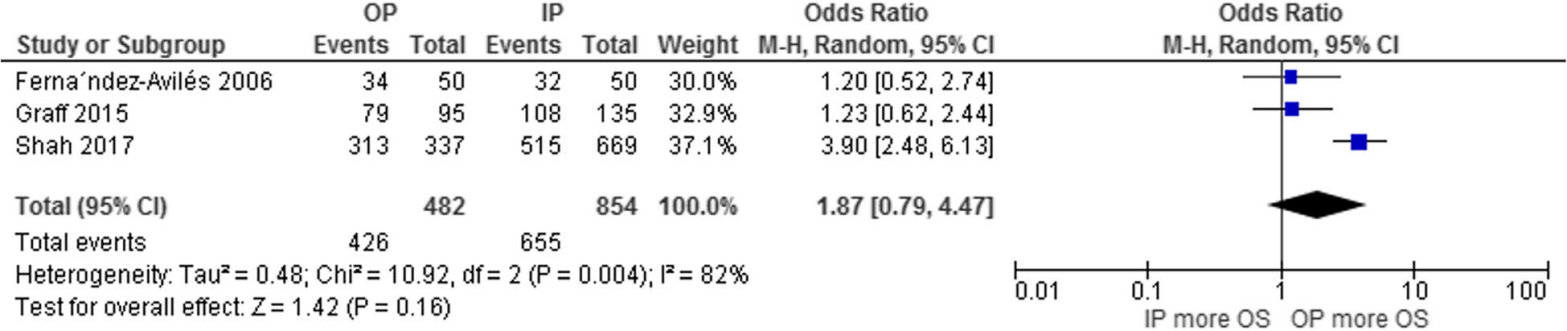
Forest plot of the analysis of the odds of surviving at 2–3 years in the outpatient versus inpatient autologous stem cell transplantation groups

The long-term OS rate was reported by three studies (the 2-year rate by two studies [26, 31], and the 3-year rate by one study [24]). Despite the odds of surviving at 2–3 years being numerically higher for those undergoing an outpatient versus an inpatient ASCT, the difference did not reach statistical significance (pooled OR 1.87; 95% CI, 0.79–4.47; *p* = 0.16; I^2^ = 82%; Fig. 5) [24, 26, 31].

### Sensitivity analysis

A sensitivity analysis was conducted by excluding the study by Fernandez-Aviles et al. [24] from the pooled analysis. This is because the main objective of the current systematic review and meta-analysis was to compare the risk of complications occurring with an outpatient versus inpatient ASCT in patients with multiple myeloma or lymphoma; however, the study by Fernandez-Aviles et al. also recruited patients with leukemia. The new pooled OR of the meta-analysis of the risk of developing febrile neutropenia increased slightly to 0.47 and remained statistically significant (95% CI, 0.32–0.69; *p* = 0.0001; I^2^ = 48%). The study by Fernandez-Aviles et al. was also included in two secondary analyses, namely, the risk of septicemia and long-term OS. However, the exclusion of that study did not significantly alter the pooled outcomes of either analysis, with a new pooled OR of 0.25 (95% CI, 0.07–0.88; *p* = 0.03; I^2^ = 18%) for the septicemia analysis and a new pooled OR of 2.26 (95% CI, 0.73–7.00; *p* = 0.16; I^2^ = 87%) for the long-term OS analysis.

### Evaluation for publication Bias

The publication bias was assessed using a funnel plot generated from the effect estimates and precision of the main analysis (risk of febrile neutropenia in the outpatient versus the inpatient group). The plot was relatively symmetric, which is not suggestive of the presence of a bias (Additional file 4: Data 3).

## Discussion

The current study is the first to comprehensively compare the risk of febrile neutropenia developing in patients with multiple myeloma or lymphoma who undergo an outpatient ASCT as opposed to an inpatient ASCT. Contrary to the conventional concerns, we found that the patients who underwent an ASCT in an outpatient setting actually had a significantly lower risk of developing infectious complications, including 56% reduced odds of developing febrile neutropenia and 60% reduced odds of developing septicemia. This observation highlights the fact that hospitalization is almost always associated with a higher risk of infection than outpatient management. An outpatient ASCT could therefore be an appealing alternative to the standard inpatient ASCT.

In addition, patients in the outpatient group were less likely to develop *C. difficile* infection, grade 2–3 mucositis, and TRM as well as more likely to survive at 2–3 years. However, the analyses for all of those outcomes did not reach statistical significance, which was partly due to the low number of eligible studies relative to the main analysis.

The popularity of outpatient ASCT is limited by the concern that the lack of the protective isolation that is usually employed during inpatient ASCT may predispose patients who underwent outpatient ASCT to a higher risk of infection. The results of the current study that demonstrate that the risk of febrile neutropenia and septicemia among patients who underwent outpatient ASCT were not higher than those who underwent inpatient ASCT (and, in fact, were lower) should help addressing this concern that hematologists could utilize this technique with more confidence.

It should also be noted that all of the included studies utilized granulocyte-colony stimulating factor (G-CSF) as a primary prophylaxis for patients in both inpatient and outpatient groups. Therefore, G-CSF primary prophylaxis has to be in the protocol if ones would apply the results of this systematic review and meta-analysis into their practices. The efficacy of G-CSF to decrease the risk of febrile neutropenia among patients with lymphoma and multiple myeloma receiving chemotherapy has been reported by multiple studies. Some selected studies on its efficacy are reviewed in Additional file 5: Table S2 [32–35]. Furthermore, the inclusion criteria for outpatient ASCT setting should also include the patient having a high educational status, the distance between the patient’s home and the hospital not being far, a good caregiver being available, the caregiver being able to take immediate action in the event of an emergency, and the provision of primary infectious prophylaxes.

However, none of the primary studies included in this meta-analysis were randomized, controlled trials. It is very likely that the characteristics of the participants in each group were different, and therefore the set of clinical variables could have been the deciding factor for clinicians when choosing an inpatient versus an outpatient strategy. This means that the observed difference in the risks of infection could be a consequence of the different baseline characteristics rather than an effect of the treatment strategy. In addition, the eligibility criteria of some of the included studies specified that patients in the outpatient group must have a good performance status as well as normal liver and renal functions [27, 30, 31]. This could have introduced a bias in the form of the selection of only healthier subjects for an outpatient arm. Moreover, one of the included studies used a less intensive conditioning regimen for outpatients than inpatients [31]. Two other limitations of this study were the moderate between-study heterogeneity of the main analysis, and the high heterogeneity of some secondary analyses.

## Conclusions

The current systematic review and meta-analysis found a significantly lower odds of developing febrile neutropenia and septicemia among patients with multiple myeloma and lymphoma who underwent an outpatient ASCT than among those who had an inpatient ASCT. This could be another appealing reason to utilize the outpatient strategy in addition to its reported lower costs and higher patient satisfaction levels. However, as the validity of the results was limited by the observational nature of the included studies, future randomized, controlled studies are still needed to confirm this potential benefit.

## Additional files

Additional file 1: **Data 1.** Search strategy. (DOCX 13 kb)

Additional file 2: **Data 2.** The Preferred Reporting Items for Systematic Reviews and Meta-Analyses statement. (DOC 63 kb)

Additional file 3: **Table S1.** ASCT procedures, infectious prophylaxis, and outpatient group management of each study. (DOCX 19 kb)

Additional file 4: **Data 3.** Funnel plot of the meta-analysis of the risk of febrile neutropenia between outpatient versus inpatient group. (DOCX 15 kb)

Additional file 5: **Table S2.** Some selected studies on efficacy of G-CSF primary prophylaxis for patients with lymphoma/multiple myeloma receiving chemotherapy. (DOCX 20 kb)

## Abbreviations

ASCT: autologous stem cell transplantation
CI: confidence interval
G-CSF: granulocyte-colony stimulating factor
HCT-CI: Hematopoietic Cell Transplantation-Comorbidity Index
OR: odds ratio
OS: overall survival
TRM: transplant-related mortality
