# Supplementary material for: Risk of febrile neutropenia among patients with multiple myeloma or lymphoma who undergo inpatient versus outpatient autologous stem cell transplantation: a systematic review and meta-analysis
Source: BMC Cancer. 2018 Nov 16;18:1126. doi: 10.1186/s12885-018-5054-6 (PMC6240267; doi:10.1186/s12885-018-5054-6)
Supplement: Supplementary file 1 — Data 1. Search strategy. (DOCX 13 kb) [file 12885_2018_5054_MOESM1_ESM.docx]

**Supplementary Data 1** Search strategy

**EMBASE**

1. 'autologous stem cell transplantation'/exp OR 'autologous stem cell transplant'
2. 'outpatient'/exp OR 'outpatient'
3. #1 AND #2

**Medline**

1. exp Transplantation, Autologous/ or exp Hematopoietic Stem Cell Transplantation/ or exp Bone Marrow Transplantation/ or autologous stem cell transplant.mp.
2. outpatient.mp. or exp outpatients/
3. 1 and 2
