# Supplementary material for: Risk of febrile neutropenia among patients with multiple myeloma or lymphoma who undergo inpatient versus outpatient autologous stem cell transplantation: a systematic review and meta-analysis
Source: BMC Cancer. 2018 Nov 16;18:1126. doi: 10.1186/s12885-018-5054-6 (PMC6240267; doi:10.1186/s12885-018-5054-6)
Supplement: Supplementary file 3 — Table S1. ASCT procedures, infectious prophylaxis, and outpatient group management of each study. (DOCX 19 kb) [file 12885_2018_5054_MOESM3_ESM.docx]

**Table S1** ASCT procedures, infectious prophylaxis, and outpatient group management of each study

| **References** | **Treatment strategy** |  |
| --- | --- | --- |
| **Morabito 2002**  **[23]** | Outpatient group | - OPD patients 45 minute driving distance of the center  - Caregiver available 24 hours-a day/7 days a week  - Admitted for PBSC infusion on day 0 for 2 days  - Availability of dedicated specialized staff and of an equipped facility operating 12 hours/day and during week-ends |
|  | Mobilization | - 3.5 to 7 g/m^2^ Cyclophosphamide in 34 cases  - VAD regimen in 7 cases  - G-CSF 5 mg/kg |
|  | Infectious prophylaxis | - Oral ciprofloxacin (500 mg every 12 hours), acyclovir (800 mg every 8 hours), fluconazole (300 mg/day) /itraconazole (200 mg/day) orally from day –5 until neutrophil recovery and trimethoprim/ sulfamethoxazole from day –8 to day 0 |
|  | G-CSF support | - 5 mg/kg/day G-CSF from 72 hours after stem cell infusion until neutrophil engraftment |
|  | Conditioning regimen | Melphalan (200 mg/m^2^/day) on days − 3 and − 2 for conditioning |
| **Ferna´ndez-Avilés 2006**  **[24]** | Outpatient group | - OPD patients 60 minute driving distance of the center  - Caregiver available 24 hours-a day/7 days a week  - Discharge on day after stem-cell infusion (day +1)  - A nurse visited the patient once daily (check temperature, blood pressure, heart frequency, presence of mucositis, oral intake, central venous catheter status, and for administering intravenous medications)  - Blood samples were taken at home three times a week |
|  | Mobilization | NR |
|  | Infectious prophylaxis | - Oral ciprofloxacin 500 mg/12 hours until neutrophil recovery, oral fluconazole 50 mg/day until day +30. |
|  | G-CSF support | - G-CSF 5 mcg/kg/d IV from day+7 until ANC reached at least 1x10^9^/L for two consecutive days |
|  | Conditioning regimen | - BEAC/M: 56, MLF 24, TBI-Cy 13, Others 7 |
| **Martino 2015**  **[25]** | Outpatient group | - OPD patients 20 minute driving distance of the center  - Caregiver available 24 hours-a day/7 days a week  - Discharged on day 1, and visited and were treated in the conventional outpatient clinic until hematological recovery  - Approval of the home by the medical staff of the bone marrow transplant unit.  (no pets at home, the sheets be changed once a day and that the home be cleaned once a day) |
|  | Mobilization | NR |
|  | Infectious prophylaxis | - Oral ciprofloxacin and acyclovir prophylaxis |
|  | G-CSF support | - 6 mg Pegfilgrastim was administered subcutaneously on day 1 |
|  | Conditioning regimen | Melphalan (200 mg/m^2^/day) on days − 3 and − 2 for conditioning |
| **Graff 2015**  **[26]** | Outpatient group | - Required daily visits with labs (comprehensive metabolic panel and complete blood count) and supportive care (including line care, fluids, transfusion) from start of conditioning until hematopoietic recovery |
|  | Mobilization | NR |
|  | Infectious prophylaxis | - Once the ANC dropped below 500/μL, oral ciprofloxacin (500 mg every 12 hours) for inpatient cohort, ertapenam intravenous once daily for outpatient  - Oral fluconazole and acyclovir prophylaxis |
|  | G-CSF support | - Single dose of pegfilgrastim on day +1 outpatient cohort , whereas the inpatient group received daily filgrastim starting on day +5, until the ANC exceeded 500/μL for 2 consecutive days |
|  | Conditioning regimen | 77 BEAM, 18 Mel140, 128 Mel200, 7 Others |
| **Paul 2015**  **[27]** | Outpatient group | - Discharged home on hospital day 3 or 4  - First outpatient follow-up visit within 2 weeks after discharge  - Home care: a visiting nurse twice a week, daily intravenous fluids and anti-emetics for 7-10 days and twice-weekly blood work |
|  | Mobilization | NR |
|  | Infectious prophylaxis | -Oral antibiotics (generally a fluoroquinolone) |
|  | G-CSF support | NR |
|  | Conditioning regimen | Melphalan (200 mg/m^2^/day) on days − 3 and − 2 for conditioning |
| **Reid 2016**  **[28]** | Outpatient group | - Caregiver available 24 hours-a day/7 days a week. Both patient and caregiver had to demonstrate an adequate level of comprehension regarding treatment plans and potential complications  - Evaluated by a nurse practitioner and attending physician on the first day of conditioning and daily thereafter by either or both.  - Laboratory monitoring included daily CBCs and chemistries |
|  | Mobilization | NR |
|  | Infectious prophylaxis | - Oral fluoroquinolone was started when the ANC dropped below 500/μL.  - Viral prophylaxis with (val)acyclovir , started with conditioning and continued for 6 months post-transplant  - Fluconazole prophylaxis, started with conditioning until engraftment |
|  | G-CSF support | NR |
|  | Conditioning regimen | BEAM-conditioning regimen |
| **Abid 2017**  **[29]** | Outpatient group | - OPD patients 30 minute driving distance of the center  - Followed up in chemotherapy daycare unit daily from D-3 till infusion day (D-0) and then three times weekly |
|  | Mobilization | Vinorelbine 25 mg/m^2^ on day 1 and cyclophosphamide 1500 mg/m^2^ on day 2 |
|  | Infectious prophylaxis | - Antibacterial prophylaxis was given from infusion onward till neutrophil engraftment or IV piperacillin–tazobactam in case of neutropenic fever  - Anti-HSV prophylaxis was started on D-2 until 3 months post transplantation  - Anti-PCP started after engraftment, until 3 months post transplantation |
|  | G-CSF support | A single dose of pegfilgrastim 6 mg on day 4 or daily doses of filgrastim 10 mcg/kg/day on day 4- 8 (at least five doses in total) |
|  | Conditioning regimen | Melphalan (100 mg/m^2^/day) on days − 3 and − 2 for conditioning |
| **Lisenko 2017**  **[30]** | Outpatient group | - OPD patients 45 minute driving distance of the center  - All daily visits and any treatment took place in the outpatient clinic, with staff previously introduced to the patients in order to avoid any stay in the waiting area to reduce the risk of infection. |
|  | Mobilization | CAD |
|  | Infectious prophylaxis | - Oral ciprofloxacin 500 mg/12 hours until haematological reconstitution  - Oral acyclovir 400 mg/ 12 hours for 6 months after ASCT |
|  | G-CSF support | NR |
|  | Conditioning regimen | Melphalan (100 mg/m2/day) on days − 3 and − 2 for conditioning |
| **Shah 2017**  **[31]** | Outpatient group | - OPD patients 30 minute driving distance of the center  - Patients <70 years old with normal organ function, committed caregiver  - Follow up the outpatient clinic 2-3 times per week until approximately 25 days after ASCT |
|  | Mobilization | NR |
|  | Infectious prophylaxis | NR |
|  | G-CSF support | NR |
|  | Conditioning regimen | Melphalan regimen or melphalan with busulfan |

**Abbreviations:** ANC: Absolute neutrophil count; ASCT: Autologous stem cell transplantation; BEAC: BCNU, etoposide, cytarabine, cyclophosphamide; BEAM: BCNU, etoposide, cytarabine and melphalan; CAD: Cyclophosphamide, doxorubicin, dexamethasone; CBC: Complete blood count; G-CSF: Granulocyte colony-stimulating factor; HSV: Herpes simplex virus; Mel Melphalan; NR: Not reported; OPD: Outpatient department; PCP: Pneumocystis carinii pneumonia; TBI-Cy: Total body irradiation-Cyclophosphamide
