# Supplementary material for: Risk of febrile neutropenia among patients with multiple myeloma or lymphoma who undergo inpatient versus outpatient autologous stem cell transplantation: a systematic review and meta-analysis
Source: BMC Cancer. 2018 Nov 16;18:1126. doi: 10.1186/s12885-018-5054-6 (PMC6240267; doi:10.1186/s12885-018-5054-6)
Supplement: Supplementary file 4 — Data 3. Funnel plot of the meta-analysis of the risk of febrile neutropenia between outpatient versus inpatient group. (DOCX 15 kb) [file 12885_2018_5054_MOESM4_ESM.docx]

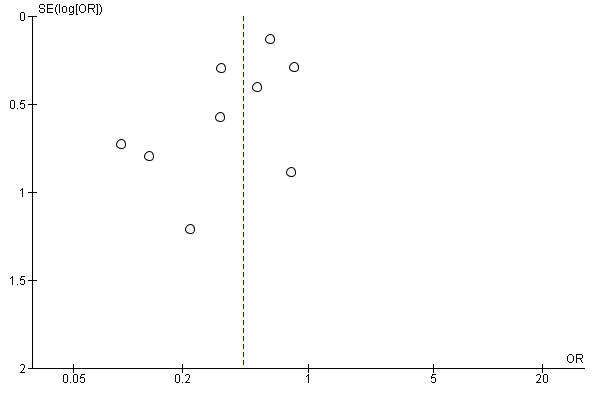


**Supplementary Data 3** Funnel plot of the meta-analysis of the risk of febrile neutropenia between outpatient versus inpatient group
