# Supplementary material for: Risk of febrile neutropenia among patients with multiple myeloma or lymphoma who undergo inpatient versus outpatient autologous stem cell transplantation: a systematic review and meta-analysis
Source: BMC Cancer. 2018 Nov 16;18:1126. doi: 10.1186/s12885-018-5054-6 (PMC6240267; doi:10.1186/s12885-018-5054-6)
Supplement: Supplementary file 5 — Table S2. Some selected studies on efficacy of G-CSF primary prophylaxis for patients with lymphoma/multiple myeloma receiving chemotherapy. (DOCX 20 kb) [file 12885_2018_5054_MOESM5_ESM.docx]

**Table S2** Some selected studies on efficacy of G-CSF primary prophylaxis for patients with lymphoma/multiple myeloma receiving chemotherapy

| **Reference** | **Patient’s group** | **Numbers** | **Disease** | **Chemotherapy regimen** | **G-CSF protocol** | **Median doses of G-CSF (range)** | **Outcome** | **Study type** |
| --- | --- | --- | --- | --- | --- | --- | --- | --- |
| Zinzani 1997  [32] | Filgrastim prophylaxis | 77 | High-grade NHL | VNCOP-B | Filgrastim (5 μg/kg/day) throughout the treatment starting on day 3 of every week for 5 consecutive days | 5 doses/week | Filgrastim primary prophylaxis was significantly associated with:  (1)Fewer incidence of grade 3 or 4 neutropenia  (2) Fewer rates of clinically relevant infections | Randomized controlled study |
|  | Control | 72 | High-grade NHL | VNCOP-B | No prophylaxis | - |  |  |
| Baldacci 2007  [33] | Pegfilgrastim prophylaxis | 73 | NHL | - CHOP  - R-CHOP  - EPOCH | Primary prophylaxis with 6 mg of pegfilgrastim  subcutaneously with a single administration on day  +1 | Single dose | Pegfilgrastim primary prophylaxis was significantly associated with:  (1) Fewer incidence of FN for patients receiving pegfilgrastim from cycle 1  (2) Fewer incidence of grade 3 or 4 neutropenia from cycle 1  (3) Fewer days of hospitalizations resulting from FN across all cycles | Randomized controlled study |
|  | Control | 73 | NHL | - CHOP  - R-CHOP  - EPOCH | No or secondary prophylaxis with pegfilgrastim in the physician-discretion | NR |  |  |
| Cerchione 2016  [34] | Pegfilgrastim prophylaxis | 24 | Relasped/refractory MM | Bendamustine-bortezomib-dexaethasone | 6 mg of pegfilgrastim  subcutaneously with a single administration on day  +4 | Single dose | Pegfilgrastim was significantly associated with:  (1) Fewer incidence rate of FN-related chemotherapy disruptions  (2) Fewer days of hospitalization due to FN  (3) Fewer G-CSF-related extrahematological  side effects | Retrospective cohort study |
|  | Control | 23 | Relasped/refractory MM | Bendamustine-bortezomib-dexaethasone | Filgrastim (5 μg/kg/day for at least 3 days), given on demand  if ANC was <1000/mm^3^ | 4.2 (3-6) |  |  |
| Cerchione 2017  [35] | Pegfilgrastim prophylaxis | 61 | Untreated indolent NHL | Bendamustine plus rituximab | 6 mg of pegfilgrastim  subcutaneously with a single administration on day  +4 | Single dose | Pegfilgrastim was significantly associated with:  (1) Fewer incidence rate of FN-related chemotherapy disruptions  (2) Fewer days of hospitalization  due to FN | Prospective cohort study |
|  | Control | 61 | Untreated indolent NHL | Bendamustine plus rituximab | Filgrastim (5 μg/kg/day for at least 3 days), given on demand  if ANC was <1000/mm^3^ | 3 (0-5) |  |  |

**Abbreviations:** ANC: Absolute neutrophil count; CHOP: Cyclophosphamide, doxorubicin, vincristine, and prednisone; EPOCH: Etoposide, doxorubicin, vincristine, cyclophosphamide, and prednisone; FN: Febrile neutropenia; G-CSF: Granulocyte-colony stimulating factor; MM: Multiple myeloma; NHL: Non-Hodgkin lymphoma; NR: Not reported; R: rituximab; VNCOP-B: Cyclophosphamide, mitoxantrone, vincristine, etoposide, bleomycin, and prednisone
